# DMSO-Free Tin Halide Perovskites for Indoor Photovoltaics

**DOI:** 10.1021/acsenergylett.5c01581

**Published:** 2025-07-14

**Authors:** Debendra Prasad Panda, Rabeb Issaoui, Zafar Iqbal, G. Krishnamurthy Grandhi, Muhammad Okash Ur Rehman, Fengshuo Zu, Paola Alippi, Madineh Rastgoo, Shengnan Zuo, Enrica Luzzi, Maxim Simmonds, Lorenzo Miele, Luigi Sanguigno, Meng Li, Paolo Aprea, Ernesto Di Maio, Norbert Koch, Paola Vivo, Antonio Abate

**Affiliations:** † Department of Chemical, Materials and Industrial Production Engineering, University of Naples Federico II, 80125 Naples, Italy; ‡ Helmholtz-Zentrum Berlin für Materialien und Energie (HZB), Hahn-Meitner-Platz 1, 14109 Berlin, Germany; § Hybrid Solar Cells, Faculty of Engineering and Natural Sciences, Tampere University, P.O. Box 541, Tampere FI-33014, Finland; ∥ CNR-ISM, Consiglio Nazionale delle Ricerche, Istituto di Struttura della Materia, Via Salaria Km 29.3, I-00015 Monterotondo Stazione, Roma, Italy; ⊥ Key Laboratory for Special Functional Materials of Ministry of Education, School of Nanoscience and Materials Engineering, Henan University, Kaifeng 475004, China; # Institut für Physik & Center for Science of Materials Berlin, Newtonstraße 15, 12489 Berlin, Germany; ¶ Department of Chemistry Bielefeld University, Universitätsstraße 25, 33615 Bielefeld, Germany

## Abstract

Indoor photovoltaic (IPV) technology has emerged as an effective strategy to sustainably power batteryless Internet of Things (IoT) devices. Though tin perovskite solar cells offer competitive IPV performance, their effectiveness is often compromised by Sn^2+^ oxidation, particularly when processed with dimethyl sulfoxide (DMSO) solvent. This work explored the IPV performance of DMSO-free tin perovskites FASnI_3–*x*_Br_*x*_ by tuning the halide composition. Notably, X-ray photoelectron spectroscopy confirms no traces of Sn^4+^, highlighting the critical role of eliminating DMSO. Under 1000 lx indoor illumination, the power conversion efficiency (PCE) increases with Br content, reaching a maximum of 11.1% for FASnI_2_Br without introducing any reducing agent. Remarkably, after six months of storage, it exhibited an impressive indoor PCE of 11.9%, demonstrating the effectiveness of the DMSO-free processing route for the intrinsic stability of the tin perovskite. These findings provide crucial insights for developing high-performance, lead-free perovskite materials for sustainable energy applications and IoT devices.

## Graphical abstract

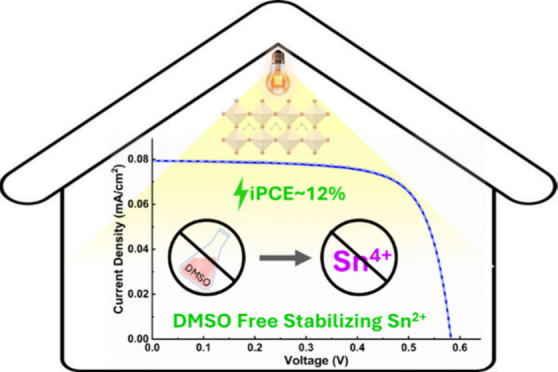

The proliferation of the Internet of Things (IoT) technologies is a cornerstone of the fourth industrial revolution. Projections estimate the deployment of one trillion interconnected devices by 2035 and a market value of several trillion dollars.^1,2^ Powering such a vast network with conventional batteries poses a major challenge due to their inherent limitations of low energy density, limited lifecycle, environmental impact, and the need for frequent replacement and maintenance.^2,3^ Indoor photovoltaics (IPV) integration offers new opportunities for seamless, autonomous, and sustainable operation of IoT devices.^4,5^ IPV converts ambient room light into electricity, where the light intensity commonly varies from 200 to 1000 lx. Notably, the emission spectrum of indoor light is narrower than that of 1-sun, spanning 380–780 nm, which necessitates the use of wide bandgap light-absorbing materials (∼1.8–2.0 eV) with bandgaps precisely tuned to match indoor illumination.^6,7^ Among the various wide bandgap materials, halide perovskites have emerged as a frontrunner, offering advantages such as tunable bandgaps, high absorption coefficients, simple and cost-effective fabrication, and excellent optoelectronic properties.^8–11^ Lead halide perovskites have recently demonstrated power conversion efficiencies (PCEs) approaching 45% under standard indoor illumination of 1000 lx.^12^ However, their toxicity and high recycling costs make them unsuitable for IoT applications. This has driven the exploration of lead-free alternatives, paving the way for developing tin-based perovskite solar cells (tin-PSCs) for IPV applications.

Though there has been a zealous effort to enhance the PCE of tin-PSCS for outdoor PV,^13–17^ the exploration of tin-PSCs for IPV application is still in its infancy. Various strategies, such as the incorporation of catechin into the precursor solution, the modification of the hole transport layer with nicotinamide and alkali metal fluorides, and the bottom interface passivation using potassium thiocyanate, have been explored for enhancing the IPV performance of tin-PSCs.^18–21^ Recently, Shakour et al. reported a PCE of 21.5% by regulating perovskite crystallization through mesomeric interactions introduced by 4-aminopyridine salt in the precursor solution.^22^ Despite these advancements, tin-PSCS still significantly lag behind their lead-based counterparts due to self-p-doping, resulting from the oxidation of Sn^2+^ to Sn^4+^.^23^ This oxidation process is notably exacerbated by the commonly used solvent dimethyl sulfoxide (DMSO).^24,25^ Therefore, employing alternative solvent systems for tin-PSCS fabrication is crucial. Our group has previously explored a solvent mixture based on N,N-diethylformamide (DEF) and N,N′-dimethylpropyleneurea (DMPU) for tin halide thin film deposition, focusing on their outdoor PV performance.^26^ However, the potential of DMSO-free systems for IPV performance of tin-PSCS remains unexplored.

As described, wide bandgap absorbers are needed to maximize IPV performance. One popular approach to enhancing the bandgap of halide perovskite is halide engineering, which can substitute iodide anions with bromide or chloride.^27,28^ Previously, Kanatzidis and co-workers have explored the structural evolution by bromine substitution and related photovoltaic properties under 1-sun illumination.^29^ By investigating the optoelectronic properties of mixed halide tin perovskites, Petrozza et al. showed that increasing bromide content reduces defect density while increasing trap state density.^30,31^ Since an increase in bromine content has a contrasting effect on defect and trap state densities and significantly increases the material’s bandgap, it is crucial to investigate how bromine concentration influences the IPV performance of tin-PSCs, which has not yet been explored.

This work explored the indoor and outdoor photovoltaic performance of DMSO-free tin perovskites through halide engineering. First, we demonstrated that the well-known self-p-doping issue in tin-PSCs can be mitigated by employing a DEF:DMPU solvent system. Subsequently, we investigated the role of lattice strain relaxation and the interplay between bandgap tuning, defect states, and trap densities on photovoltaic performance. Our findings show that compositions with low Br content achieved a maximum PCE of 8.1% under 1-sun illumination. In contrast, high Br content perovskite with wider bandgap exhibited a state-of-the-art indoor efficiency of 11.1% under 1000 lx illumination. Notably, this performance was achieved without employing any reducing agents or antioxidants, underscoring the intrinsic quality of the material. Importantly, the devices demonstrated exceptional long-term stability with a PCE of 11.9% after 6 months of storage, highlighting the effectiveness of the DMSO-free solvent system in enabling sustainable IPV applications.

To investigate the role of bromine substitution, we prepared a series of tin perovskites, FASnI_3–*x*_Br_*x*_, where *x* = 0, 0.2, 0.5, 0.8, and 1. The perovskite precursor solution was prepared using a DMSO-free solvent system composed of DEF and DMPU, as mentioned in our earlier report.^26,32^ We performed X-ray photoelectron spectroscopy (XPS) on the tin perovskites processed with a DMSO-free solvent mixture to examine the chemical state, as shown in Figure 1a. The Sn 3*d* core levels of parent FASnI_3_ samples exhibit no trace of Sn^4+^, confirming the complete suppression of Sn^2+^ oxidation. In contrast, the same FASnI_3_ sample, which was subsequently exposed to air for 2 h, exhibits a rigid shift of Sn 3d core levels by 1.25 eV toward higher binding energy, clearly evidencing the oxidation into the Sn^4+^ state. We also note that upon increasing the Br content, the Sn 3d core levels shift slightly toward higher binding energy by 0.29 eV, likely due to the enlargement of the bandgap as discussed in subsequent sections. Notably, no SnF_2_ additives or antioxidants were used in the precursor solution, confirming that the suppression of Sn^2+^ oxidation primarily stems from using this alternative solvent system.

**Figure 1:**
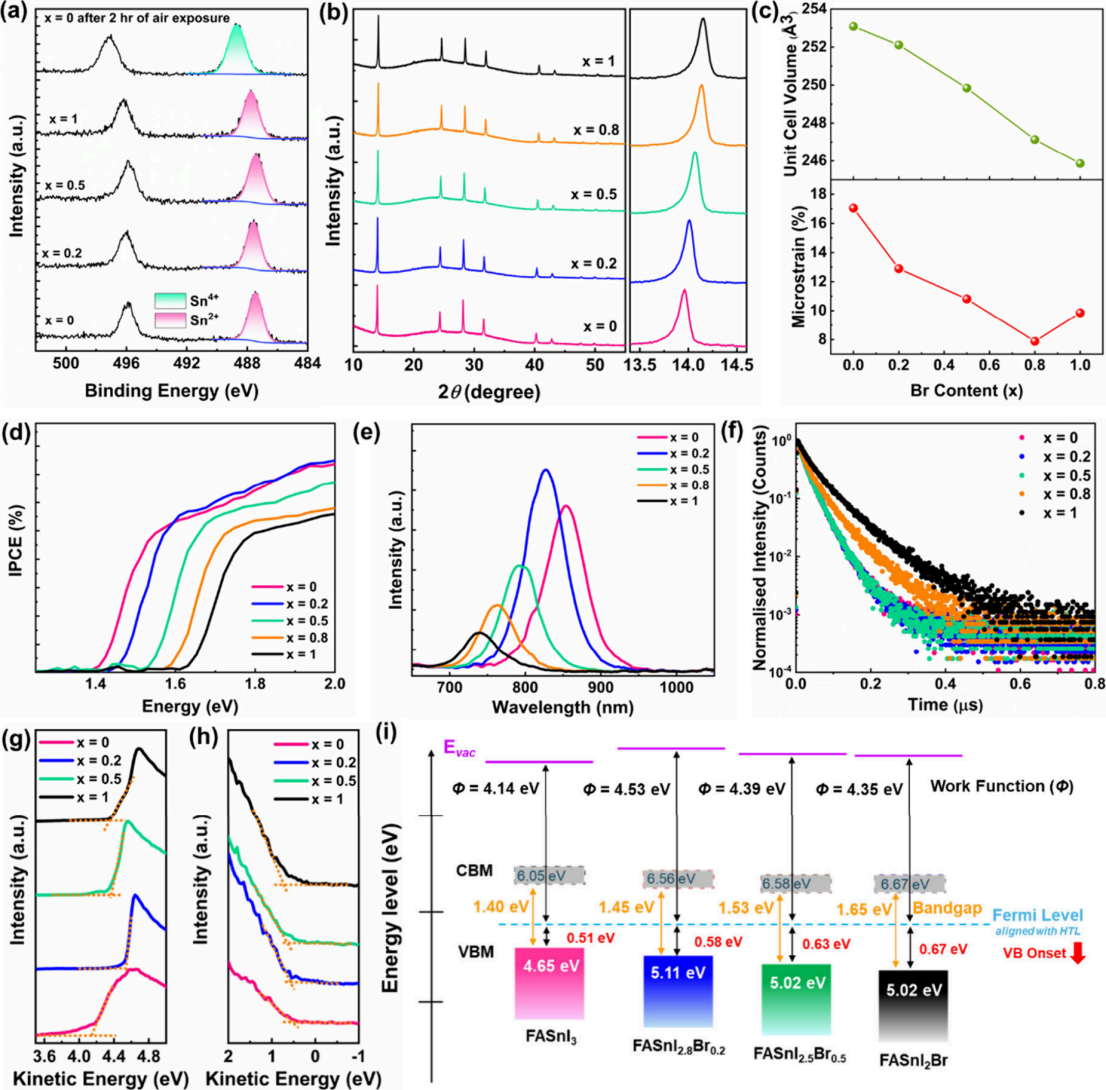
Effect of DMSO-free solvent and Br-substitution on Sn^2+^ oxidation, structural, optical, and electronic properties: (a) High-resolution Sn 3*d* spectra of DMSO-free tin perovskites with different Br content indicating the absence of the Sn^4+^ oxidation state (emergence of Sn^4+^ states at higher binding energy after 2 h air exposure is shown for contrast). (b) X-ray diffraction pattern and (c) variation of unit cell volume and microstrain of the tin perovskites at different Br content. (d) Bandgap of the FASnI_3–*x*_Br_*x*_ PSCs. (e) Steady-state photoluminescence spectra exhibit a nonmonotonic intensity trend. (f) Time-resolved PL spectra indicate improved charge carrier dynamics and suppression of the recombination rate. (g) Secondary electron cutoff (SECO) region, (h) valence spectra, and (i) energy band diagram from energy level measurements of FASnI_3–*x*_Br_*x*_ (*x* = 0–1) samples. The Fermi level at a 0 eV binding energy is aligned with the hole transport layer.

The structural parameters with different Br contents were studied using X-ray diffraction (XRD). The parent compound, FASnI_3_ (*x* = 0), crystallizes in a cubic structure with the *P*m-3m space group.^33^ Upon Br incorporation, the crystal structure remains unchanged, with no additional peaks, confirming the phase purity (Figure 1b). However, a peak shift toward higher 2θ values is observed, signifying a reduction in d_*hkl*_-spacing, which can be attributed to the smaller ionic radius of Br^–^ (1.96 Å) compared to I^–^ (2.2 Å). As a result, the unit cell volume *V*, evaluated from a Le Bail analysis (Figure S1), linearly decreases from 253.09 Å^3^ (*x* = 0) to 245.86 Å^3^ (*x* = 1), consistent with Vegard’s law for solid solutions (Figure 1c).^33,34^ Additionally, the full width at half-maximum (fwhm) of the (100) peak decreases with increasing Br content (Table S2), indicating an improvement in crystallinity.^35^ The enhanced crystallinity can reduce the lattice microstrain (*ε*), which is quantified through the Le Bail fitting of the XRD patterns (Figure 1c). The microstrain decreases with increasing bromine content, reaching a minimum value of 7.9% for *x* = 0.8, almost 1 order of magnitude lower than the parent FASnI_3_ sample. This reduction in microstrain can be attributed to a decrease in crystal lattice defects, leading to improved lattice ordering.^36,37^ A further increase in bromine content results in a slight rise in macrostrain, although it remains significantly lower than that of the *x* = 0 sample. In addition to the reduction in crystal defects, we believe that the observed decrease in microstrain with increasing bromine content may also be attributed to the formation of a more symmetrical structure, as indicated by the tolerance factor calculations.^38^ A tolerance factor closer to 1 corresponds to an ideal cubic perovskite structure. In this case, the tolerance factor increases linearly from 0.978 (*x* = 0) to 0.984 (*x* = 1), suggesting a gradual shift toward higher structural symmetry, possibly leading to strain relaxation in the material (Figure S2). Further, the top-view images of scanning electron microscopy demonstrate that the average grain size ranges from 281 ± 92 nm for *x* = 0 to 294 ± 60 nm for *x* = 1 (Figure S3). Although the incorporation of bromine induces minimal changes in the microstructure, the *x* = 1 sample displays more uniform and well-ordered grains, supporting the XRD finding.

Halide engineering in perovskites is a facile approach for tuning the bandgap of these compounds. Figure 1d illustrates the optical bandgap variation with changing I:Br ratios in tin perovskites, determined from the onset of the respective devices’ incident photon-to-current efficiency (IPCE). The optical bandgap for the *x* = 0 sample is 1.4 eV and increases with higher bromine content, reaching 1.65 eV for *x* = 1. The nearly linear increase in bandgap is consistent with Vegard’s law for solid solutions and corresponds to the gradual decrease in unit cell size or interatomic distance (Figure 1c). The bandgap values were supported by UV–vis absorption spectroscopy (Figure S4), which showed excellent agreement with the IPCE data.

To further investigate the effect of halide engineering on the optical properties and defect chemistry of tin perovskites, we performed steady-state photoluminescence (PL) spectroscopy. As expected, the PL peak position exhibited a blue shift with increasing Br content, confirming bandgap widening and consistent with the optical bandgap of the samples (Figure 1e). However, the PL intensity followed a nonmonotonic trend with increasing Br content. The emission peak intensity was initially enhanced as the Br content increased from *x* = 0 to 0.2, while a further increase in Br content led to a gradual decline in PL intensity. This nonlinear trend can be understood by analyzing the interplay of *p*-doping and trap states, which significantly influence PL intensity in perovskite materials. *p*-doping can promote radiative monomolecular charge recombination, thereby amplifying PL intensity.^39^ In contrast, trap states reduce PL intensity by facilitating nonradiative charge carrier decay.^40^ Meanwhile, bromination in tin perovskites decreases overall defect density and increases trap densities.^41^ This should typically result in a linear decrease in PL intensity with an increasing Br content. However, the bromination-induced dedoping can slow down Auger recombination, a nonradiative process in which excess energy is transferred to a third charge carrier.^39,41^ So, possibly, slowing down this Auger recombination can enhance the PL intensity and the nonmonotonous trend of PL intensity resulting from the interplay of defects, trap densities, and Auger recombination. Such a nonlinear trend was earlier observed in MASn­(I_1–*x*_Br_*x*_)_3_ perovskite for higher Br content.^31^ Furthermore, the full-width at half-maximum (fwhm) of the emission peak increased from 0.11 to 0.14 eV as *x* increased from 0 to 1, likely due to enhanced self-trapping of charge carriers.^42–44^ Additionally, time-resolved photoluminescence (TRPL) measurements demonstrate a continuous increase in the average carrier lifetime from 31.3 ns (*x* = 0) to 59.8 ns (*x* = 1), indicating suppressed recombination losses (Figure 1f). This further supports the decrease in defect density and increase in carrier trapping with rising Br content.

To examine the impact of halide engineering on the energy level of tin perovskites, we have performed ultraviolet photoelectron spectroscopy (UPS) on FASnI_3–*x*_Br*_*x*_* (*x* = 0–1) samples. The work function (WF) values are directly determined from the secondary electron cutoff region (SECO) in Figure 1g: WF = photon energy – (binding energy of the SECO). Figure 1h shows the valence band (VB) spectra of these samples on a linear intensity scale of the photoelectrons, where the VB maximum values are extrapolated.^45,46^ The corresponding energy levels of the samples are presented in Figure 1i and Table S3, where the conduction band minimum values are extracted, given the optical band gaps determined in Figure 1d. We observed that upon increasing the Br content, the sample exhibits a slight increase of VB maximum from 0.51 eV for *x* = 0 to 0.67 eV for *x* = 1, in line with the rise of the Sn 3d core levels toward higher binding energy. This is accompanied by an increase in the sample work function upon Br incorporation, leading to a rise in ionization energy from 4.65 eV for *x* = 0 to 5.02 eV for *x* = 1. This lowering in VBM can be attributed to the Br *p*-orbital’s lower energy than I, driven by its higher electronegativity. In contrast, the conduction band minimum (CBM) shifts upward due to the upward shift of Sn p-orbitals as Br substitutes. We can relate to the shortening of the bond between Sn–Br, which causes an increase in Sn-confined electrons’ energy, leading to an upward shift of the CBM and a downward shift of the VBM, thereby increasing the band gap.^27,47^ We speculate that lowering the VB onset away from the Fermi level is beneficial for achieving a higher open-circuit voltage (*V*oc) due to improved energy level alignment with the hole transport layer.^48^

Further, we have explored the effects of Br substitution on equilibrium volume, local geometries, and band structure of FASnI_3_, by performing Density Functional Theory (DFT) calculations on a set of configurations with mixed I/Br halide composition (Details in the Supporting Information). The changes in the gap, valence, and conduction band positions obtained *ab initio* are compared with those of experiments in Figure 2a. Typically, shrinking the lattice constant in halide perovskite lowers the band gap. Here, this effect only partially offsets the band gap increase due to chemical I/Br substitution, leading to an overall increase rate of E_g_ in agreement with the experimental one (Table S4). In Figure 2a, theoretical (DFT, blue empty spheres, dashed line) and experimental (expt, blue filled squares) levels are aligned to the corresponding FASnI_3_ valence band top, and a constant shift has been applied to the DFT conduction band to correct for the well-known underestimation of the theoretical band gap. Furthermore, our calculations for V_Sn_ (relaxed V_Sn_ configuration is shown in Figure S6), the dominant native acceptor in FASnI_3_, show that its formation energy increases by 0.1 eV between *x* = 0 and *x* = 1, leading to a drop in its concentration by a factor of around 60 at room temperature. This corresponds to an upward Fermi level shift of about 0.1 eV in the same I/Br compositional range.

**Figure 2:**
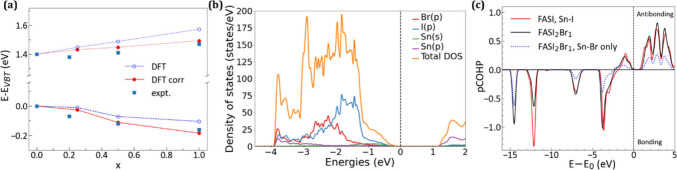
DFT calculations: (a) DFT (circles) and experimental (squares) band edge positions (eV) as a function of Br content. Values are aligned to the corresponding valence band top for *x* = 0. Calculated DFT values were corrected by a linear Fermi energy shift (filled circles). (b) Partial density of states (pDOS, arbitrary units) for FASnI_2_Br. (c) pCOHP for FASI and FASnI_2_Br. The dotted line shows the pCOHP for Sn–Br interactions in FASnI_2_Br. Energy zero is the respective valence band top.

Next, we discuss the projected density of states (pDOS) and Crystal Orbital Hamiltonian Population (COHP)^49^ calculated from the DFT wave functions with the LOBSTER code^50^ and shown in Figure 2b and 2c, respectively. In the valence region between −4 and 0 eV, the I states in bulk FASnI_3_ are centered around −1.5 eV (Figure S7). In FASnI_2_Br, the pDOS shows a transfer of spectral weight toward lower energies in the same region (Figure 2b). Approximately one-third of the anion states originate from Br and are centered around – 2.5 eV, while the I states remain at – 1.5 eV but with a reduced contribution of two-thirds compared to the full iodide contribution in FASnI_3_. The COHP augments this description by visualizing the bonding or antibonding character of the interaction between specific atoms as a function of the electronic energy. Figure 2c displays the pCOHPs for the Sn–I interaction in FASnI_3_ and the sum of Sn–Br and Sn–I interaction in FASnI_2_Br in the valence energy range as well as the pCOHP for Sn–Br-only interactions. The downward shift of the pCOHP weight for FASnI_2_Br compared to FASnI_3_ signals an increase in the bonding character in the occupied states, and the Sn–Br pCOHP indicates the key role of Br in downshifting spectral weight and energy eigenvalues, as suggested by the pDOS. The 2.5% calculated change in ICOHP between them confirms the decrease in the antibonding character of the valence band induced by Br substitution.

The devices were fabricated with the *p-i-n* configuration with the device architecture ITO/PEDOT-complex/Al_2_O_3_/FASnI_3–*x*_Br_*x*_/C_60_/BCP/Ag, where *x* varies from 0 to 1 (Figure 3a). The forward and reverse *J*–*V* scans of champion devices of each composition are depicted in Figure 3b. The photovoltaic parameters are listed in Table S5, and the statistics are shown in Figure 3d-g. All of the *J–V* curves show negligible hysteresis, indicating high-quality devices under bias stress. The parent FASnI_3_ (*x* = 0) shows open-circuit voltage (*V*_*OC*_) of 0.58 V, short-circuit current density (*J*_*SC*_) of 19.2 mA/cm^2^, fill factor (FF) of 67% and the power conversion efficiency (PCE) of 7.4%. With an increase in the Br content, the *V*_*OC*_ gradually increases, and a maximum *V*_*OC*_ of 0.72 V is achieved for FASnI_2_Br (*x* = 1) composition. This increment in *V*_*OC*_ predominantly results from the increase in bandgap as confirmed from the UV–vis and UPS spectra.^29,41^ In addition, lower defect densities result in higher *V*_*OC*_.^51^ It is worth noting that the relaxation of microstrain and improved microstructure at higher Br levels indicate lower defect density. In the meantime, *J*_*SC*_ decreases continuously as the Br level increases and becomes 11.6 mA/cm^2^ for *x* = 1 sample. This reduction was further corroborated by IPCE spectra (Figure 3c), where the integrated current density closely matched the values obtained from the *J–V* measurements. The observed decrease in *J*_*SC*_ can be attributed to various factors. First, the overall light absorption decreases with a higher Br incorporation (Figure S8), reducing the photocurrent generation. Also, it is possible that the energy loss can be increased due to bandgap widening.^52^ Furthermore, the increase in trap densities with increasing Br content hampers charge extraction, further contributing to the diminution of *J*_*SC*_. As a result of this contrasting trend of *V*_*OC*_ and *J*_*SC*_, the PCE follows a nonmonotonic trend. The PCE increases from 7.4% (*x* = 0) to 8.1% for the *x* = 0.2 composition with *V*_*OC*_, *J*_*SC*_, and FF of 0.61 V, 19.0 mA/cm^2^ and 70%, respectively. However, further Br incorporation leads to a decline in PCE. This variation in PCE with Br content closely mirrors the trend observed in PL intensity, highlighting the intricate interplay among crystal lattice modifications, bandgap evolution, defect densities, and trap states. Furthermore, the dark *J–V* scans (Figure 3h) indicate that the leakage current of the devices minimizes with increasing Br content, which correlates well with the corresponding *V*_*OC*_ trend. To ensure the stability of the devices, PCE was measured under maximum power point tracking (MPPT) conditions in a N_2_-filled glovebox, revealing superior stability of *x* = 0.2 compared to other compositions (Figure 3i). However, the devices with higher Br content degrade faster under 1 sun, possibly due to ion migration or the enhanced trap densities.

**Figure 3:**
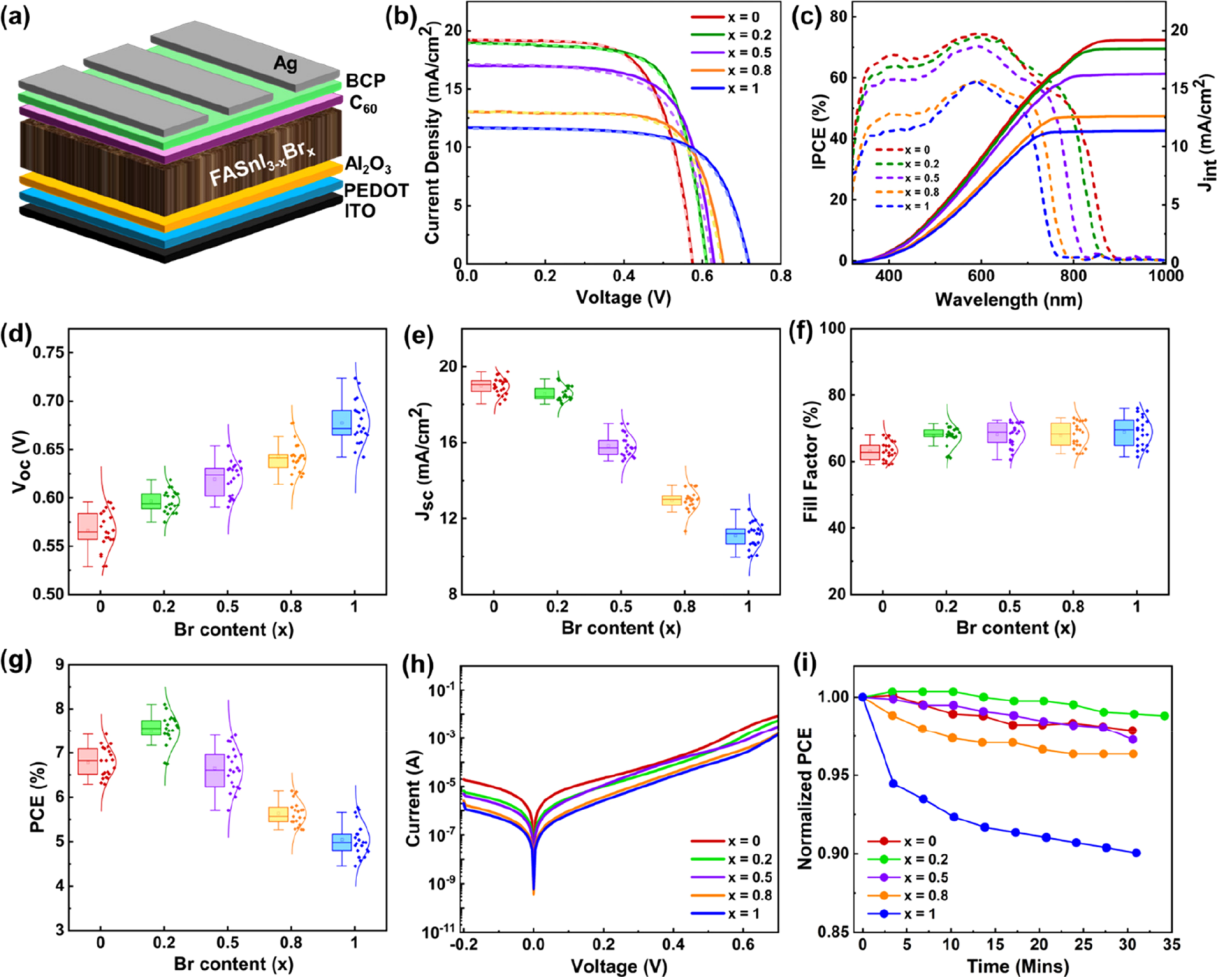
Photovoltaics performance under AM 1.5G illumination: (a) Schematic diagram of device architecture. (b) *J–V* curves and (c) IPCE data of the champion device of each composition of FASnI_3–*x*_Br_*x*_ perovskites at different Br levels. The statistics of (d) *V*_*OC*_, (e) *J*_*SC*_, (f) FF, and (g) PCE of FASnI_3–*x*_Br_*x*_ perovskites. (h) Dark *J–V* curves indicate reduced leakage current with increasing Br levels. (i) MPP tracking shows that tin perovskite with a low Br content of *x* = 0.2 has higher device stability.

Finally, we have explored the IPV performance of the DMSO-free tin perovskite under white light-emitting diodes (WLEDs) with different color temperatures (CTs) and intensities. Figure 4a compares the spectra of AM 1.5G and warm white LEDs (2900 and 3600 K), highlighting the lower intensity and narrower spectral range (400–740 nm) of indoor light, which necessitates the use of wide bandgap absorbers for effective indoor harvesting. First, we measured the IPV performance of the tin perovskites with different Br content under 1000 lx WLED illumination. The J–V scans for the champion device and the corresponding parameters of each composition are shown in Figure 4b and Table S6, respectively. FASnI_3_ (*x* = 0) shows a PCE of 8.6% with *V*_*OC*_, *J*_*SC*_, and FF values of 0.34 V, 0.1 mA/cm^2^, and 69%, respectively. The *J*_*SC*_ decreases and *V*_*OC*_ increases with the increase in the Br content, similar to the photovoltaic performance under 1-sun. However, unlike the PCE trend under 1-sun, which exhibited a nonmonotonic increase with increasing Br level, the PCE under 1000 lx demonstrates a continuous rise with Br content and reaches a maximum value of 11.1% with *V*_*OC*_, *J*_*SC*_, and FF of 0.58 V, 0.08 mA/cm^2^, and 72%, respectively for FASnI_2_Br (*x* = 1). The mismatch between IPCE and J–V is less than 10% (Figure S9), showing the authenticity of our indoor measurements. Notably, the PCE of 11.1% is one of the highest for tin perovskite IPV devices without any reducing agents or antioxidants.^18,19^ While the variations in *J*_*SC*_ and FF are not significant, the *V*_*OC*_ of the devices gradually and significantly increases with Br content from 0.36 (*x* = 0) to 0.58 V (*x* = 1), indicating the pivotal role of the band gap of the tin perovskite absorber in determining the IPV performance. While *x* = 0.2 is the optimal composition under 1-sun, *x* = 1 performs better under indoor lighting conditions. Due to its wider bandgap and blue-shifted absorption edge, the *x* = 1 composition exhibits better spectral overlap with indoor light sources compared to the *x* = 0.2 composition (Figure S10), which can contribute to the improved PCE of *x* = 1 under indoor illumination. However, lower photocurrent of *x* = 1 composition, resulting from its reduced absorbance across the 400–750 nm range (Figure S8) indicate that spectral matching alone does not account for the improved indoor PCE. Instead, the enhanced efficiency of the *x* = 1 device under low-light conditions is primarily attributed to reduced recombination losses, including bimolecular recombination, as evidenced by the improved *V*_*OC*_ and FF.^4^ Notably, *V*_*OC*_ increases from 0.34 V (*x* = 0.2) to 0.58 V (*x* = 1) with only a 0.2 eV increase in bandgap (1.45 to 1.65 eV), indicating a reduction in voltage loss at higher Br content. These results underscore the critical role of voltage retention and reduced carrier recombination losses for efficiency gains under indoor illumination, where carrier generation is intrinsically limited.

**Figure 4:**
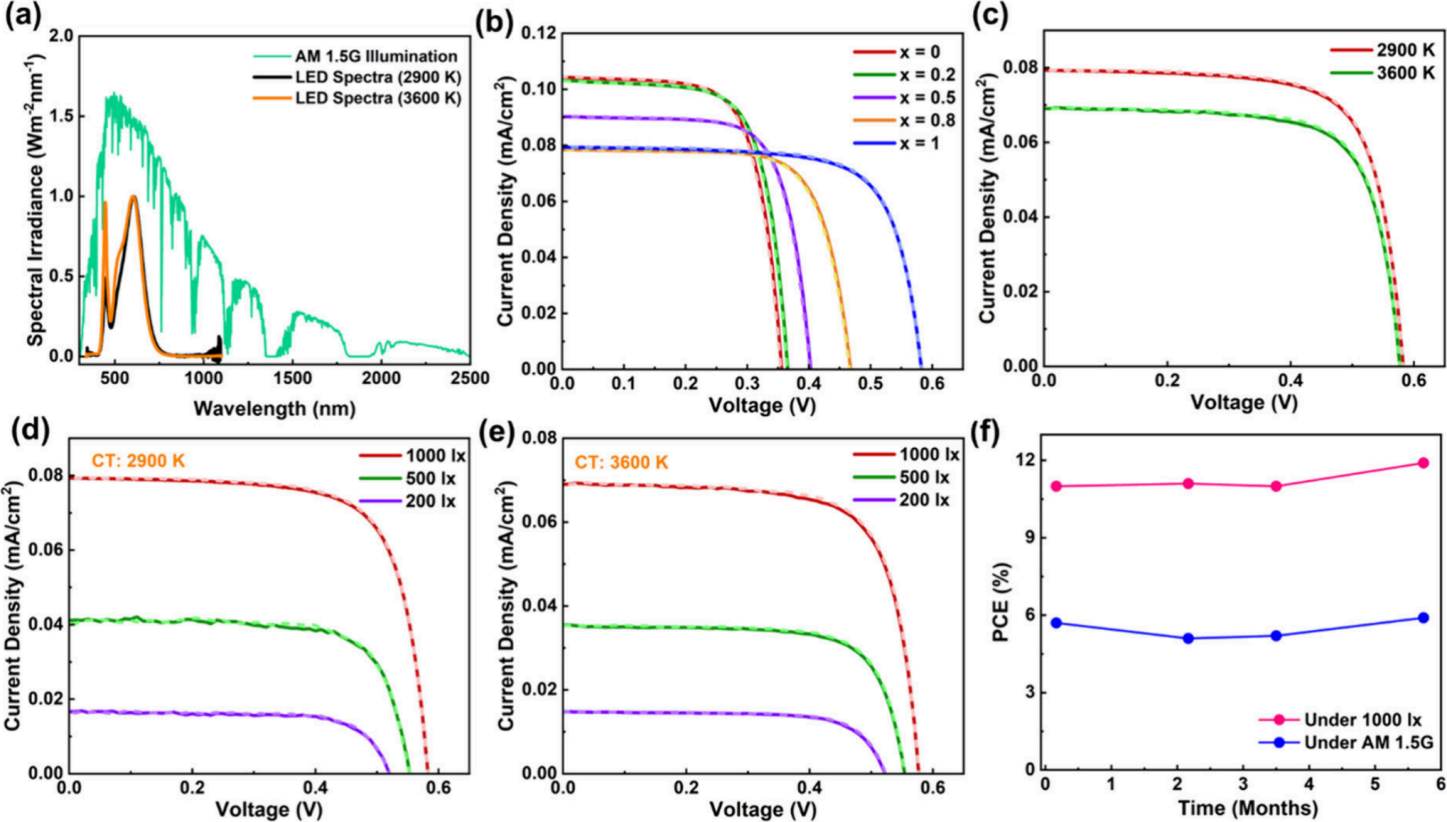
Indoor photovoltaics performance of FASnI_3–*x*_Br_*x*_ perovskites: (a) Comparison of AM 1.5 G and LEDs emission spectra with different color temperatures (2900 and 3600 K). (b) *J–V* curves of the champion device of each composition of FASnI_3–*x*_Br_*x*_ perovskites under 1000 lx illumination. (c) *J–V* curves of the champion device of FASnI_2_Br under LEDs with different CT. *J–V* curves under various light intensities of LEDs of (d) 2900 and (e) 3600 K CT. (f) PCE variation of champion FASnI_2_Br device under N_2_ atmosphere for up to 6 months, measured under AM 1.5 G and 1000 lx illumination.

We further explored the effect of LED color temperature (CT) on the IPV performance of the FASnI_2_Br device under 2900 and 3600 K WLED illumination, and the corresponding *J–V* response is presented in Figure 4c. Under 2900 K illumination, the device achieved a PCE of 11.1%, which declined to 9.6% as the CT increased to 3600 K. This reduction in the PCE is primarily attributed to a decrease in *J*_*SC*_. As CT increases, the emission spectra of the LED light shift toward shorter wavelengths (Figure 4a), resulting in a more significant spectral mismatch between the LED emission and the absorption spectrum of FASnI_2_Br, which has a band edge around 760 nm. Consequently, the reduced spectral overlap limits light absorption, resulting in a lower *J*_*SC*_ and a corresponding decline in overall PCE. Considering diverse indoor application scenarios, we also investigated the IPV performance under different light intensities. As the illumination intensity decreases from 1000 to 200 lx, the PCE drops from 11.1% to 10.0% and 9.6% to 8.9% under 2900 and 3600 K WLEDs illumination, respectively (Figure 4d-e and Table S7). This decline is primarily attributed to the reduced number of photogenerated charge carriers under weaker illumination, which weakens the open-circuit voltage *V*_*OC*_ and *J*_*SC*_.^53,54^ The linear decrease in *J*_*SC*_ with decreasing light intensity suggests that the ratio of incident photons to converted electrons remains constant. In contrast, the reduction in *V*_*OC*_ is more pronounced due to an increased influence of trap-assisted recombination under weak light conditions.^53^ The *V*_*OC*_ losses under low light intensity can be understood using the equation: $V_{OC}=V_{OC,ref}+\frac{nk_{B}T}{q}\ln\left(\frac{I}{I_{ref}}\right)$ , where *V*_*oc,ref*_ is the *V*_*OC*_ under 1000 lx, *n*, *k*_*B*_, *q* refer to ideality factor, Boltzmann constant, and electron charge, respectively.^55^ The extracted ideality factor (*n*) from this fitting is 1.587 (Figure S11), indicating that Shockley–Read–Hall recombination predominantly governs the junction current.^56^ Consequently, *V*_*OC*_ (along with FF) is a dominant factor affecting device efficiency at low light intensities, highlighting the need for effective defect passivation strategies and controlling the crystallization dynamics to further enhance performance under indoor lighting conditions.

The devices were stored in a nitrogen-filled glovebox to assess the stability of the DMSO-free tin perovskites. Notably, the FASnI_2_Br device improved PCE to 11.9% under 1000 lx illumination after 6 months of storage, demonstrating remarkable long-term stability (Figure 4f). This is the first report on the IPV stability of tin perovskites under storage conditions. This enhanced stability was observed not only under indoor lighting conditions but also under 1-sun illumination, highlighting the robustness of the mixed-halide tin perovskite. We attribute this exceptional stability to the absence of DMSO in the precursor solution, which likely mitigates the degradation pathways typically associated with tin perovskites. This finding underscores the potential of DMSO-free Sn perovskites for long-term applications in IoT devices.

In conclusion, we systematically investigated the impact of halide engineering on the structural, optical, electronic, and outdoor and indoor photovoltaic properties of DMSO-free tin perovskites, FASnI_3–*x*_Br_*x*_. First, our study demonstrates that replacing DMSO with a DEF:DMPU solvent system results in no or negligible Sn^2+^ oxidation, addressing a key limitation of tin-based PSCS. Second, increasing the Br ratio leads to better microstructure, lattice strain relaxation, bandgap widening, and improved charge carrier dynamics. Under one sun illumination, the PCE follows a nonmonotonic trend with the rising Br level from *x* = 0 to 1, showing the maximum PCE of 8.1% for *x* = 0.2, highlighting the complex interplay of bandgap tuning, strain relaxation, defect, and trap states. In contrast, under standard indoor lighting of 1000 lx, the PCE increases continuously with Br content, reaching 11.1% for *x* = 1, mainly due to the reduced defect density and increased bandgap. The device demonstrated relatively stable IPV performance under varying CT and intensities of WLED lighting. Remarkably, after 6 months of storage, a state-of-the-art indoor PCE of 11.9% (1000 lx) was achieved, demonstrating the exceptional stability of these DMSO-free devices. These findings offer valuable insights for advancing high-efficiency and stable lead-free perovskite solar cells optimized for low-light energy harvesting, paving the way for sustainable IoT applications.

## Supplementary Material



## Notes

Detailed experimental methods (device and film fabrication and their characterization); DFT calculation details; Le Bail fitting of XRD patterns; tolerance factor, SEM images, and UV–vis absorbance spectra of mixed halide tin perovskites; relaxed lattice structure and density of states; *J*_*SC*_ mismatch between IPCE and *J*–*V* measurement; spectral matching of WLED spectrum with IPCE spectra of tin perovskites; variation of *V*_*OC*_ under different light intensities; and supporting tables (PDF)
