# Supplementary material for: DMSO-Free Tin Halide Perovskites for Indoor Photovoltaics
Source: ACS Energy Lett. 2025 Jul 14;10(8):3789–98. doi: 10.1021/acsenergylett.5c01581 (PMC12341655; doi:10.1021/acsenergylett.5c01581)
Supplement: Supplementary file 1 [file nz5c01581_si_001.pdf]

## Supplementary Information

# DMSO-Free Tin Halide Perovskites for Indoor Photovoltaics

Debendra Prasad Panda[1], Rabeb Issaoui[1], Zafar Iqbal[2], G. Krishnamurthy Grandhi[3], Muhammad Okash Ur Rehman[1], Fengshuo Zu[2], Paola Alippi[4], Madineh Rastgoo[1], Shengnan Zuo [2], Enrica Luzzi[1], Maxim Simmonds[2], Lorenzo Miele[1], Luigi Sanguigno[1], Meng Li[5], Paolo Aprea[1], Ernesto Di Maio[1], Norbert Koch [2,6], Paola Vivo[3]*, and Antonio Abate[1,2,7]*

[1]*Department of Chemical, Materials and Industrial Production Engineering, University of Naples Federico II, 80125 Naples, Italy.*

[2]*Helmholtz-Zentrum Berlin für Materialien und Energie (HZB), Hahn-Meitner-Platz 1, 14109 Berlin, Germany.*

[3]*Hybrid Solar Cells, Faculty of Engineering and Natural Sciences, Tampere University, P.O. Box 541, Tampere FI-33014, Finland.*

[4]*CNR-ISM, Consiglio Nazionale delle Ricerche, Istituto di Struttura della Materia, Via Salaria Km 29.3, I-00015 Monterotondo Stazione, Roma, Italy.*

[5]*Key Laboratory for Special Functional Materials of Ministry of Education, School of Nanoscience and Materials Engineering, Henan University, Kaifeng 475004, China.*

[6]*Institut für Physik & Center for Science of Materials Berlin, Newtonstraße 15, 12489 Berlin*

[7]*Department of Chemistry Bielefeld University, Universitätsstraße 25, 33615 Bielefeld, Germany.*

### Corresponding Author

* Antonio Abate

Email: antonio.abate@unina.it, antonioabate83@gmail.com

* Paola Vivo

Email: paola.vivo@tuni.fi

**Materials and Devices**

**Chemicals:**

ITO was purchased from Ossila B.V. Water-free PEDOT complex dispersion in toluene (HTL3) was obtained from CleviosTM. Formamidinium iodide (FAI) and Formamidinium brominde(FABr) were purchased from Dyenamo. Silver shot (1–5 mm, 99.9%) was supplied by Alfa Aesar, and $C_{60}$ was procured from CreaPhys. All other chemicals, including $SnI_2$, DEF (N,N-Diethylformamide), DMPU (N,N′-Dimethylpropyleneurea), toluene, $Al_2O_3$, ethanol, diethyl ether (DEE), Bathocuproine (BCP), and ethylenediammonium diiodide ($EDAI_2$) were obtained from Sigma-Aldrich and used without further purification.

**Device Fabrication:**

The patterned ITO substrates were cleaned using Hellmanex solution, deionized (DI) water, acetone, and isopropyl alcohol. After thorough drying, the substrates underwent UV-ozone treatment for 15 minutes. Immediately following the UV-ozone treatment, the cleaned substrates were transferred into a nitrogen-filled glovebox. It is important to note that all fabrication steps were conducted within a high-purity nitrogen-filled glovebox that had never been exposed to dimethyl sulfoxide (DMSO). Solvent regeneration was frequently performed to prevent solvent vapor accumulation. PEDOT complex was spin-coated onto the substrates at 4000 rpm. The films were then annealed at 150 °C for 10 minutes. Then $Al_2O_3$ nanoparticles were spin-coated at 4000 rpm for 40 secs and anneled at 100 °C for 5 minutes The stock solutions of $FASnI_3$ and $FASnI_2Br$ were prepared in a DMSO-free solvent system consisting of N,N-diethylformamide and N,N'-dimethylpropyleneurea (DEF:DMPU, 6:1 v:v) using $SnI_2$ (1.2 M), FAI (1 M) and FABr (1M). The $FASnI_{3-x}Br_x$ (x = 0.2, 0.5, and 0.8) precursor solution was prepared by dissolving the stoichiometric amount of $FASnI_3$ and $FASnI_2Br$ solutions. Then the perovskite ink (with 5% $EDAI_2$) was spin-coated at 4000 rpm for 40 seconds. After 22 seconds of spinning, 100 μL of diethyl ether (DEE) was dropped onto the substrate to induce crystallization, followed by annealing at 100 °C for 30 minutes. As the electron selective contact, $C_{60}$ and BCP were sequentially evaporated under a vacuum of $10^{-6}$ mbar. Silver was then evaporated through a shadow mask. The final device area was approximately 0.17 $cm^2$. The Devices were stored in the glovebox until measurement.

**Film Characterization:**

**X-ray diffraction (XRD):** Powder X-ray diffraction (XRD) was performed using the PANalytical instrument (Cu K$\alpha_1$ and K$\alpha_2$ radiation) fitted with a PIXcel1D detector. We have used the film stackas Glass/PEDOT-complex/$Al_2O_3$/$FASnI_{3-x}Br_x$.

**Scanning Electron Microscopy (SEM):** SEM measurement was performed on the top surface of the films (ITO/PEDOT-complex/$Al_2O_3$/$FASnI_{3-x}Br_x$) using FEI Quanta 200 FEG instrument.

**UV-VIS-NIR Absoprtion Spectroscopy:** Absorption spectrum was performed by Cary Series UV-Vis-NIR spectrophotometer from Agilent Technologies under double beam mode. The perovskite films (Glass/PEDOT-complex/$Al_2O_3$/$FASnI_{3-x}$Br) were encapsulated by a thin cover glass before measuring in the air.

**Photoluminescence (PL) Spectroscopy:** PL spectra were recorded inside the glovebox using a commercial platform (Arkeo-Ariadne, Cicci Research s.r.l.). The substrate was illuminated at a 45° angle with a diode-pumped solid-state Nd/$YVO_4$ + KTP laser (peak wavelength 532 ± 1 nm, optical power 1 mW on a 2 mm diameter circular spot, resulting in an intensity of 31 $mWcm^{-2}$). Fluorescence from the illuminated side of the substrate was focused onto a 10 mm diameter fiber bundle using an aspheric lens positioned near the substrate to optimize PL collection. The signal was then transmitted to a CCD-based spectrometer for analysis. The integration time and averaging parameters were kept consistent to allow for better comparison of the results. We have used the film stacking as Glass/PEDOT-complex/$Al_2O_3$/$FASnI_{3-x}Br_x$ for this measurement.

**Time Resolved Photoluminescence (trPL)**: Performed with a home-built confocal PL setup in air on encapsulated samples. The setup featured a “80:20”- “transmission:reflection”-beam splitter to separate the excitation and detection paths. For excitation, a SuperK FIANIUM white light laser source was used, with an excitation window of 545-555nm at 153kHz rep rate. The laser beam was passed through a 650nm shortpass filter (FESH650, Thorlabs). The energy of the laser beam was tuned by a linear-gradient neutral density filter to ≈2 µW. An off-axis parabolic mirror with 5 cm focal length has been used for the focus and PL collection. The laser spot size is a circle with ≈120 µm diameter. A silicon single-photon avalanche diode (Laser Components COUNT50) has been employed for the PL detection, and the signal was cut by a 650 nm long-pass filter (FELH650, Thorlabs). The PL count and decay histogram were recorded by a TimeHarp260 Nano time-correlated single photon counting module (Picoquant).

**Ultraviolet and X-ray Photoelectron Spectroscopy (XPS and UPS):** Ultraviolet photoelectron spectroscopy measurements were performed using a monochromatized helium discharge lamp (photon energy at 21.22 eV) in an ultrahigh vacuum system (base pressure at 1 × $10^{-9}$ mBar), acquired using a hemispherical SPECSPhoibos 100 analyer. The UV flux was attenuated by a factor of 100 as compared to the standard helium lamp to reduced irradiation induced degradation. X-ray photoelectron spectroscopy measurements were conducted using a monochromatized Al Kα radiation (1486.6 eV) using a JOEL-9030 XPS system. The secondary electrons cutoff (SECO) was acquired with a sample bias at -10 V. All spectra were acquired at room temperature and normal emission. The overall energy resolution was set at 110 meV and 800 meV for UPS and XPS, respectively.

**Device Characterization:**

J-V curves were recorded using a commercial system (Arkeo-Ariadne, Cicci Research s.r.l.) equipped with a ring of 12 LEDs covering wavelengths from 300 to 1000 nm, calibrated to meet class A standards, and utilizing a 4-wire source meter. The incident photon-to-current efficiency (IPCE) spectrum was measured using the same commercial system (Arkeo-Ariadne, Cicci Research s.r.l.), which employs a 300-Watt xenon lamp to capture the spectrum across the 300 to 1000 nm range. For indoor photovoltaic measurement, was measured using the same Arkeo set up with two different white light LEDs.

**DFT Calculations:**

We have performed *ab initio* Density Functional Theory (DFT) calculations within the plane-waves, projector augmented wave (PAW) method[1] implemented in the Vienna ab initio simulation package (VASP).[2–6] Total energy calculations have been performed within the generalized gradient approximation (GGA) with the Perdew-Burke-Ernzerhof parametrization for exchange-correlation interaction.[7] We have included DFT-D3 dispersions.[8] DFT calculations for $FASnI_{3-x}Br_x$ compositions have been carried out in 2x2x2 supercells of rotated $FASnI_3$ cubic unit cell (16 octahedra). The wavefunctions cut-off has been set at 500 eV, and the Brillouin zone has been sampled by a (2x2x2) grid. We have considered $FASnI_{3-x}Br_x$ compositions with x = 0, 0.25, 0.5, 0.7 and 1, corresponding to Br/I atoms ratios in the supercells equal to 0/48, 4/44, 8/40, 12/36, 16/32. For the x=0.2 composition, we generated 5 equivalent configurations obtained by randomly replacing I atoms with Br ones (**Figure S4**) and we calculated ground state energy after atomic forces minimization (up to calculated forces

< 0.03 eV/A): the more dispersed configuration is energetically favored, although the electronic structure characteristics (band gap, bands edges shifts) do not change appreciably. The DFT band edge levels have been aligned internally to the average electrostatic potential at Sn cores. In the calculations, we account for volume changes due to Br substitution, which leads to an approximate 1% decrease in lattice constant when x=1 (**Table S4**).

For each I/Br composition, we performed three sets of calculations where the lattice constant $a$ was either optimized (values of $a_{eq}$ for each x are reported in Table S1), or fixed at the experimental or theoretical $a_{FASI}$. Table S1 reports the band gap values, along with the variations of the valence band top (VBT) and conduction band bottom (CBB). The latter were calculated by aligning the calculated values to the average electrostatic potential at Sn cores. At fixed volume, the chemical substitution of I with Br lowers the valence band edge and increases the conduction band bottom. This results in a band gap increase by +380 meV when going from x=0 to x=1 in the $FASnI_{3-x}Br_x$ formula unit (columns 6,7). The optimized volumes decrease as Br content increases: here, this effect does not totally offset the band gap increase due to Br inclusions, with $E_g$ increasing by +277 meV (column 5).

**Figures:**

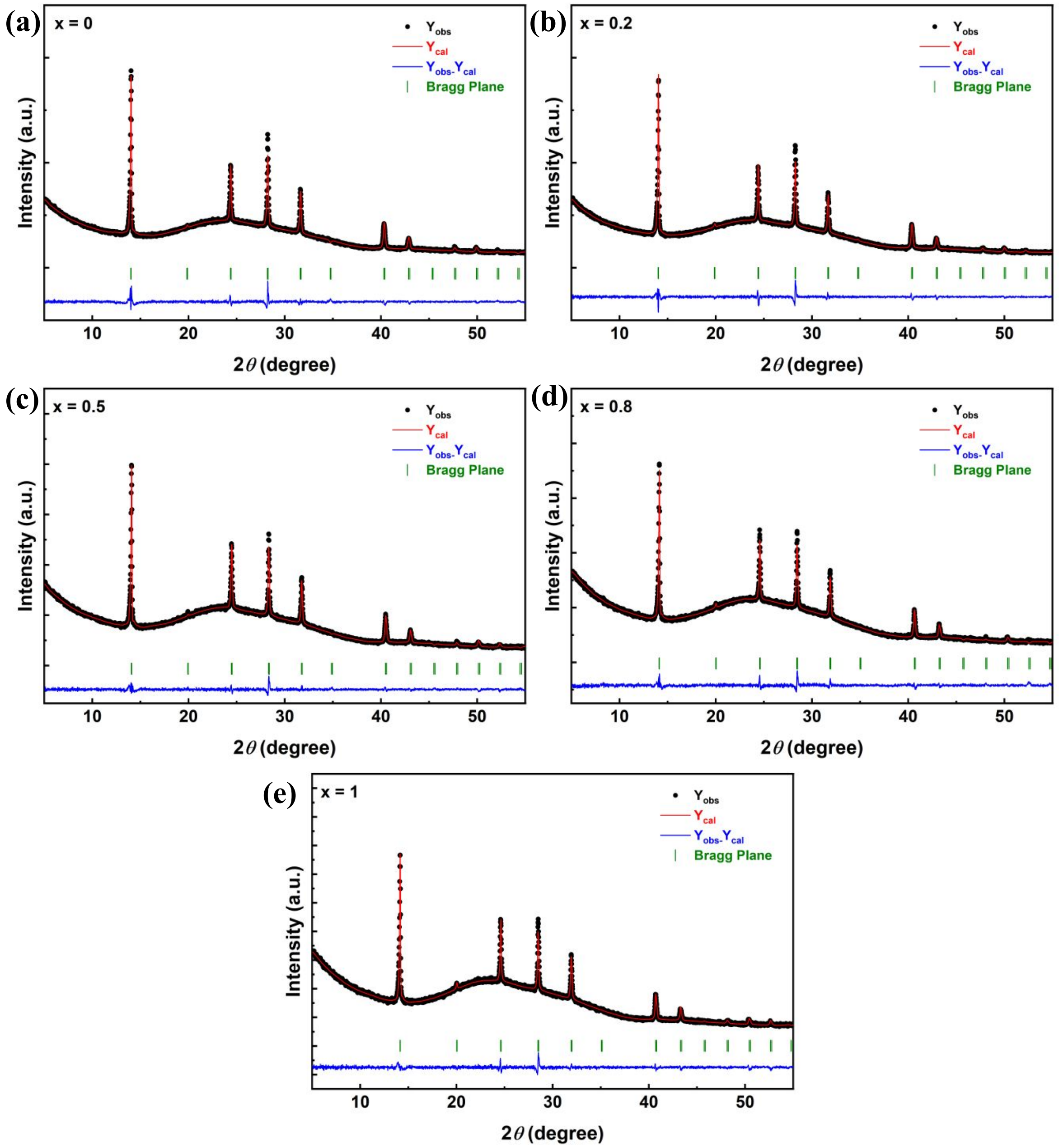


**Figure S1.** Le Bail refinement of x-ray diffraction pattern of $FASnI_{3-x}Br_x$ ($0 \leq x \leq 1$) perovskites.

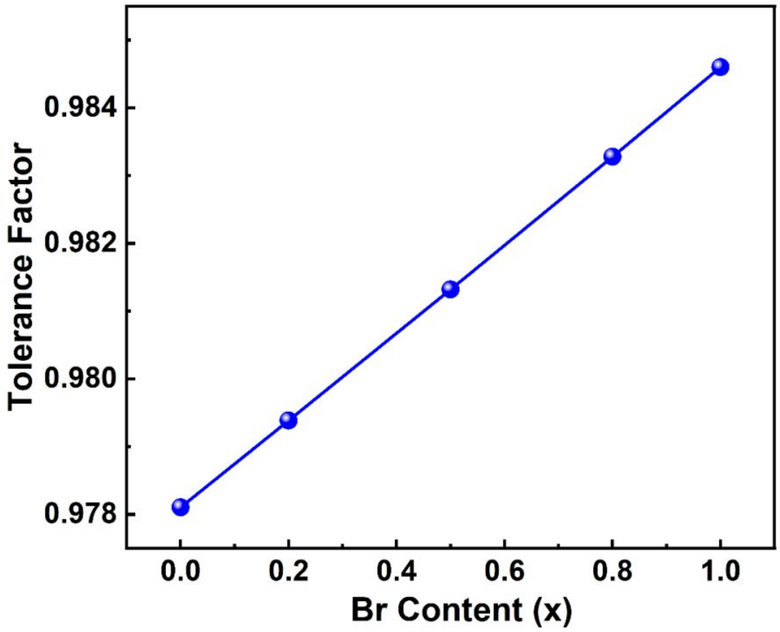


**Figure S2.** Variation of tolerance factor with increasing Br content in $FASnI_{3-x}Br_x$ perovsites.

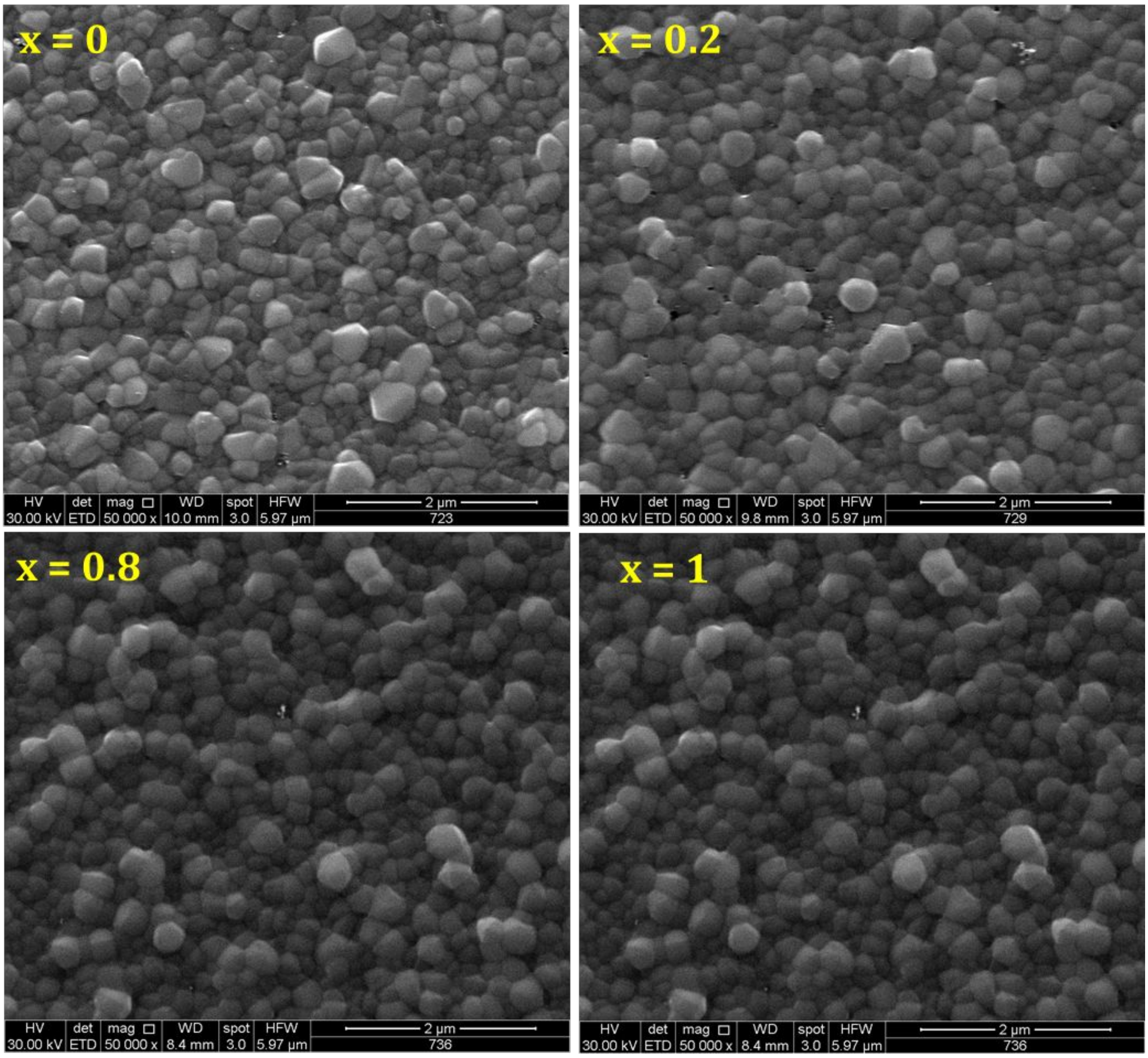


**Figure S3.** Top view SEM images of $FASnI_{3-x}Br_x$ perovskites.

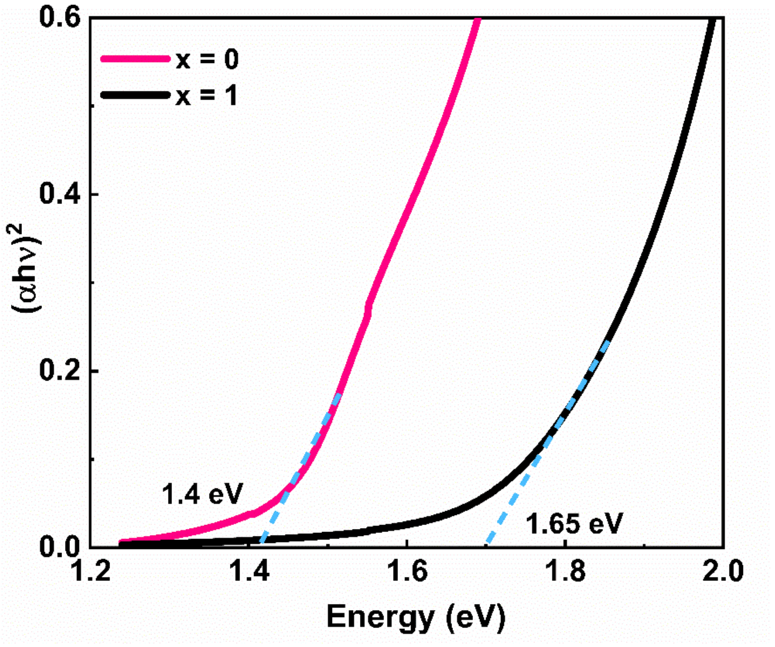


**Figure S4.** Tauc plot for bandgap calculation obtained from the UV-Vis spectroscopy of $FASnI_{3-x}Br_x$ perovskites.

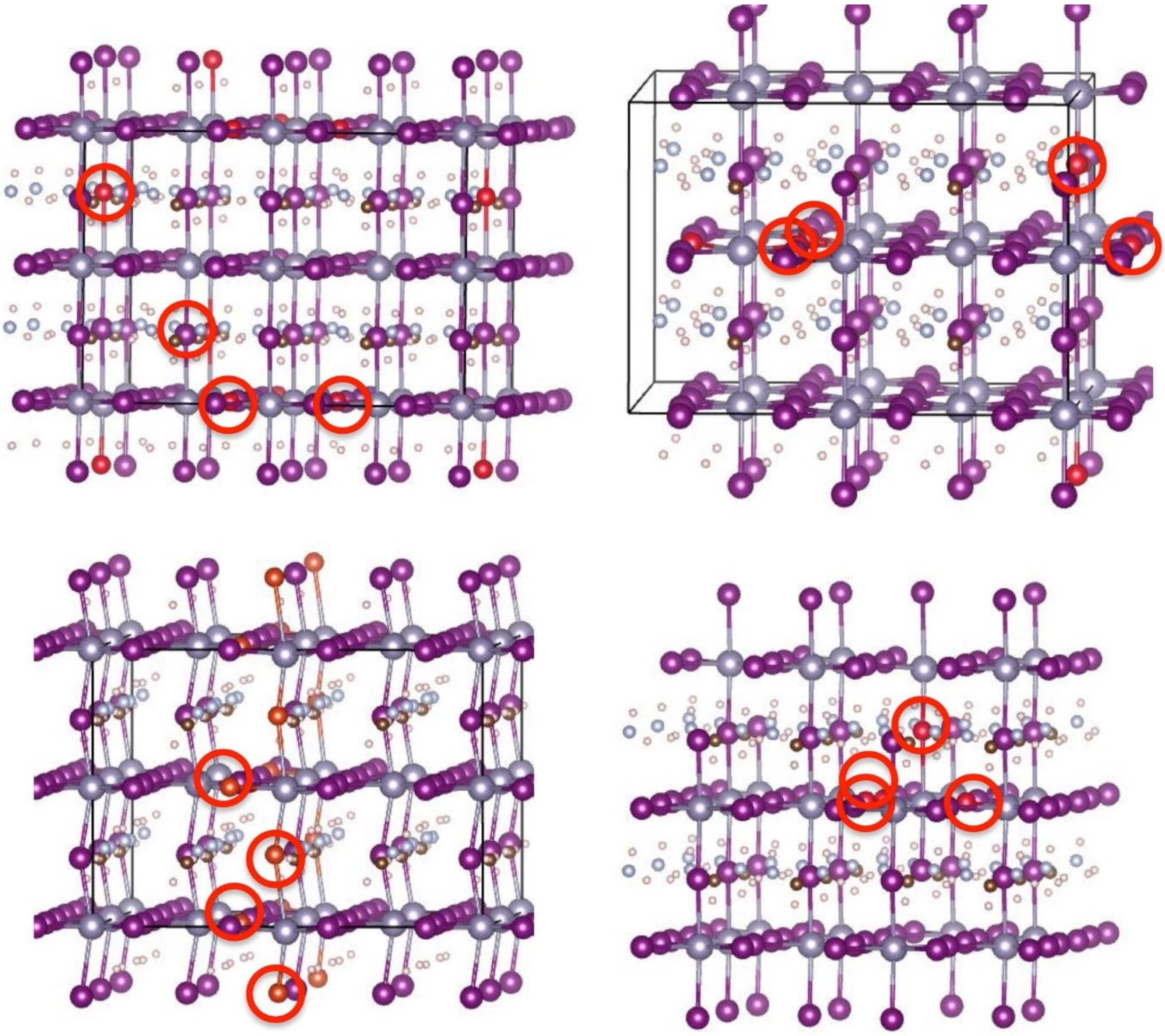

**Figure S5.** Atomic configurations of equivalent configuration for $FASnI_{2.75}Br_{0.25}$. Red circles highlight the position of inequivalente Br atoms in the unit cell.

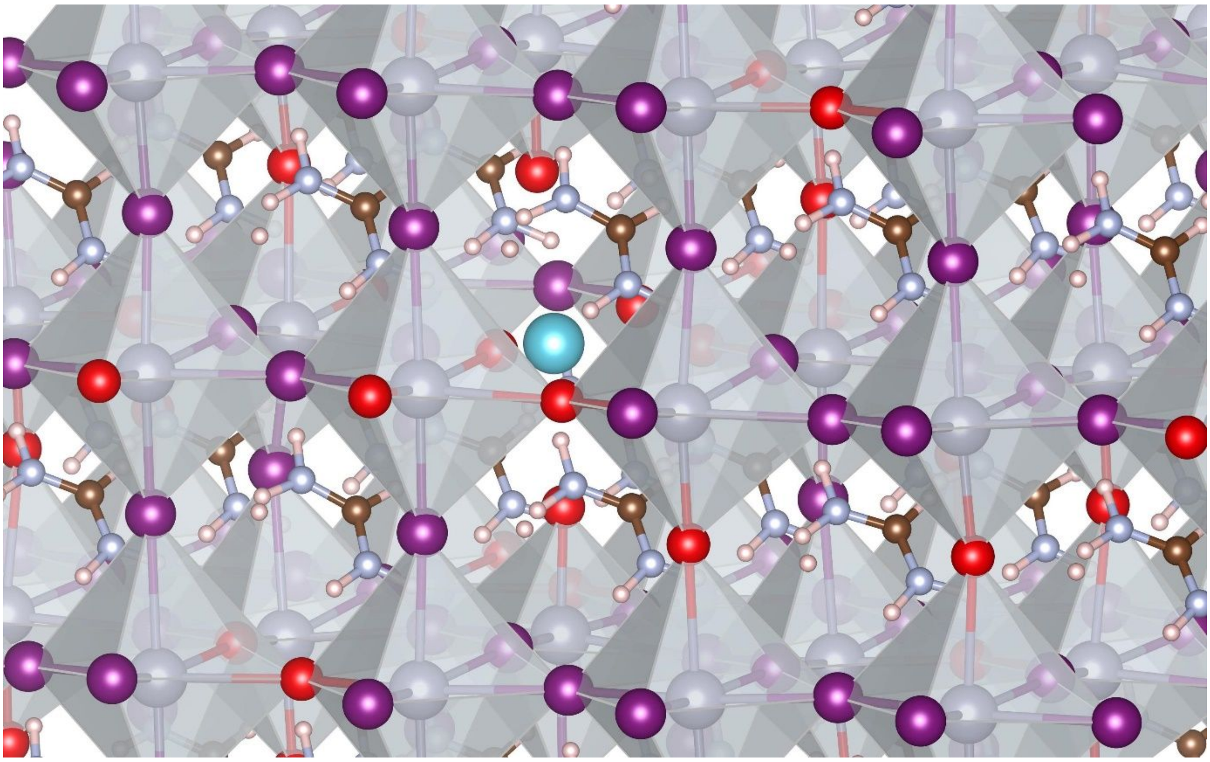

**Figure S6.** Relaxed configuration for the neutral Sn vacancy defect $V_{Sn}$ in the $FASnI_2Br$ supercell. The initial position of missing Sn atom is highlighted in light blue.

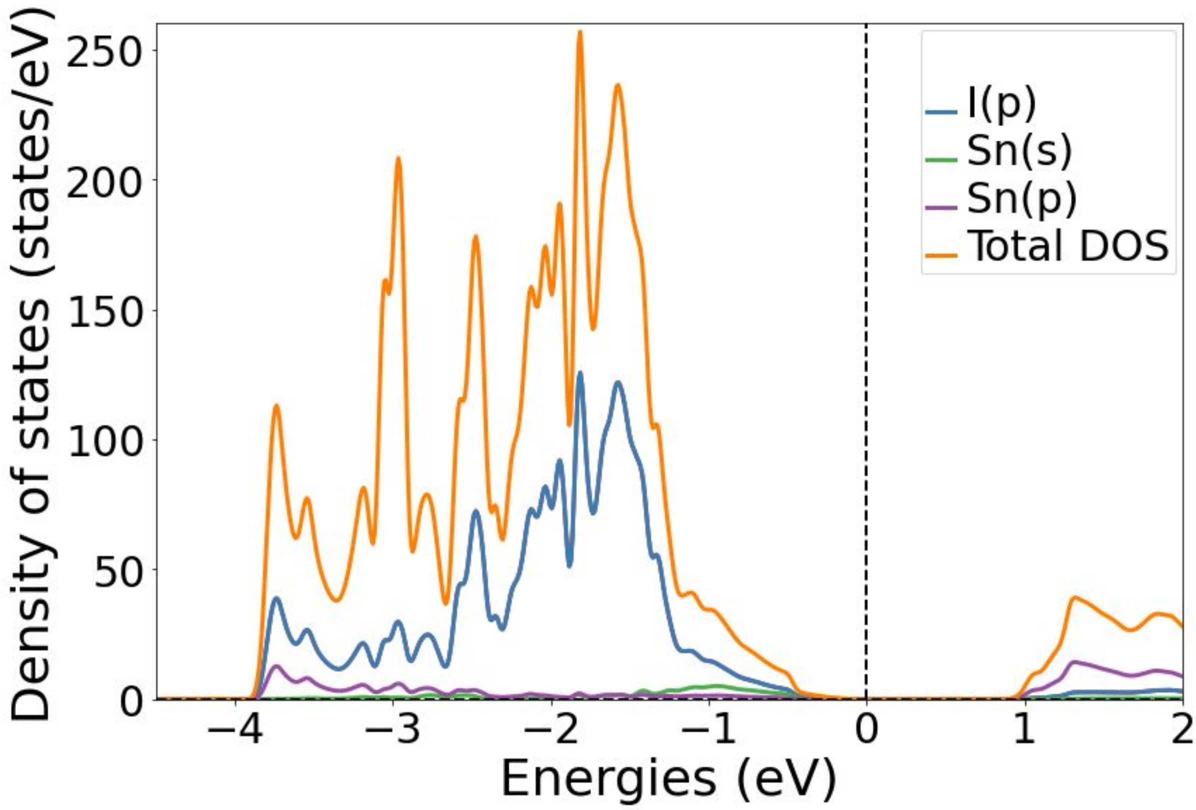


**Figure S7.** Partial density of states (pDOS, arbitrary units) for FASnI. I-*p* (blue line) and Sn-*s*, *p* (green and violet line). Total DOS is also shown as orange line.

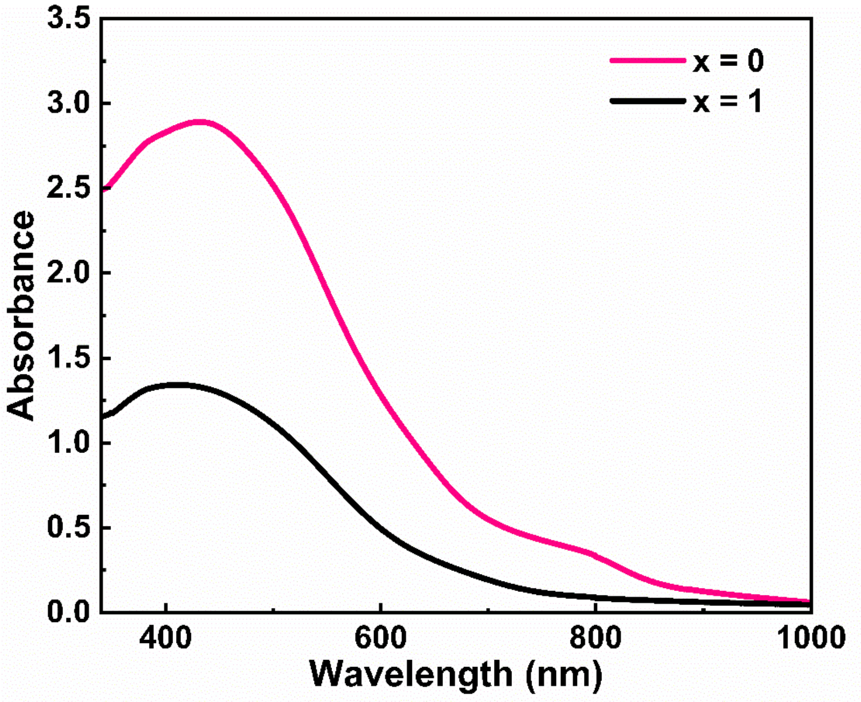


**Figure S8.** Absorbance spectra of $FASnI_{3-x}Br_x$ perovskites, where $x = 0$ and 1.

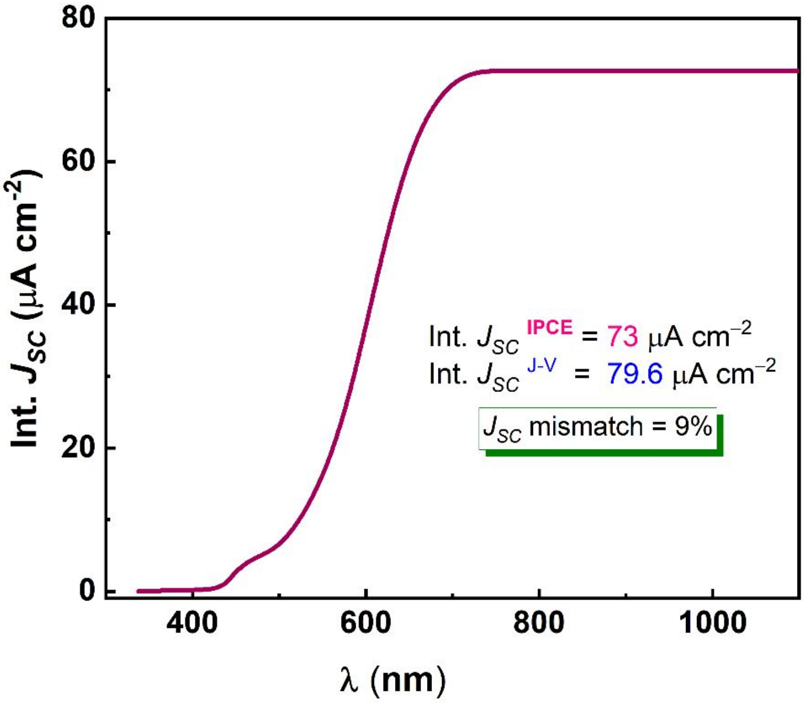


**Figure S9**. Mismatch between the $J_{SC}$ obtained from IPCE and J-V measurement is below 10%.

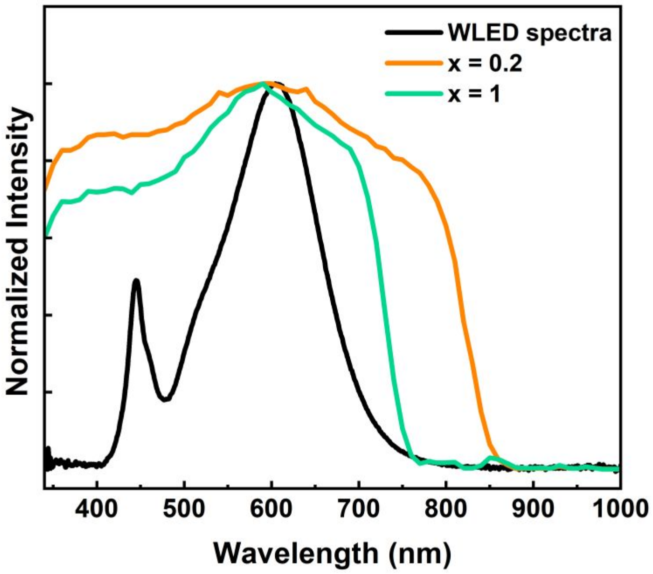


**Figure S10**. IPCE spectra of the $x = 1$ composition, showing a blue-shifted photoresponse onset and improved spectral matching with the WLED emission spectrum compared to the $x = 0.2$ composition. Each spectrum is normalized to facilitate comparison of spectral matching.

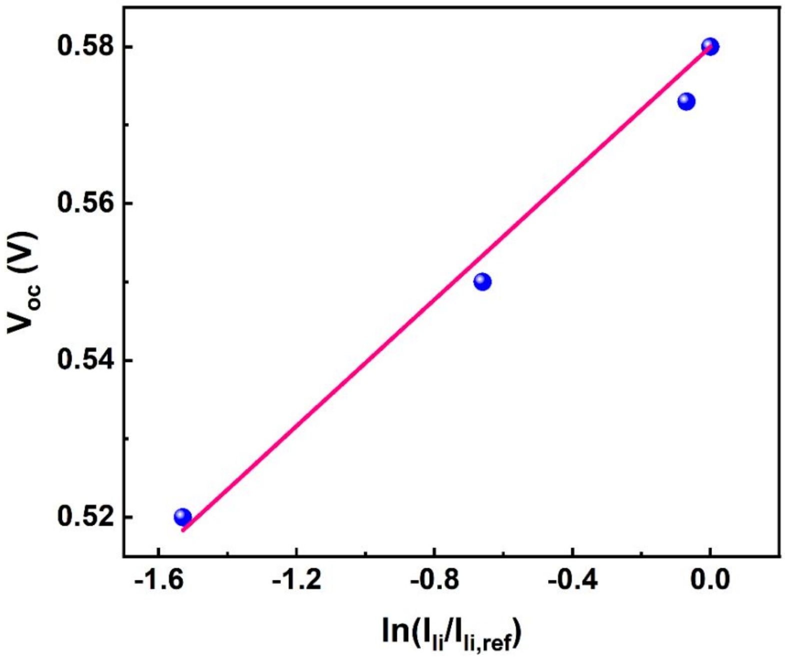


**Figure S11.** Variation of $V_{OC}$ with different light intensity under low light condition for $FASnI_2Br$ composition.

**Tables:**

**Table S1.** The calculated stoichiometry for all samples are very close to the values of the precusors.

| **STD-Xray** | **I $3d_{3/2}$** | **Br $3d_{5/2}$** | **I/Br**<br>**Atomic ratio** | **I/Br**<br>**Per solution** |
|---|---|---|---|---|
| RSF | 12.59 | 1.55 | | |
| $FASnI_{2.8}Br_{0.2}$ | 1962.4 | 18.163 | 13.3 | 14 |
| [Effective] | [155.8] | [11.7] | | |
| $FASnI_{2.5}Br_{0.5}$ | 2082.0 | 55.217 | 4.6 | 5 |
| | [165.3] | [35.6] | | |
| $FASnI_2Br$ | 1919.2 | 115.8 | 2.0 | 2 |
| | [152.4] | [74.7] | | |

**Table S2.** Full-width at half maxima of the (100) XRD peak of $FASnI_{3-x}Br_x$ ($0 \leq x \leq 1$) perovskites.

| **Br content ($x$)** | **FWHM (º)** |
|---|---|
| 0 | 0.1195 |
| 0.2 | 0.1172 |
| 0.5 | 0.1153 |
| 0.8 | 0.1151 |
| 1 | 0.1117 |

**Table S3.** The values of WF, VBM (valence band maximum), and CBM (conduction band minima) for $FASnI_{3-x}Br_x$ perovskites.

| Sample | Band gap (eV) | WF/ev | VB onset | Ionization Energy (eV) | CBM |
|---|---|---|---|---|---|
| $FASnI_3$ | 1.40 | 4.14 | 0.51 | 4.65 | 6.05 |
| $FASnI_{2.8}Br_{0.2}$ | 1.45 | 4.53 | 0.58 | 5.11 | 6.56 |
| $FASnI_{2.5}Br_{0.5}$ | 1.53 | 4.39 | 0.63 | 5.02 | 6.58 |
| $FASnI_2Br$ | 1.65 | 4.35 | 0.67 | 5.02 | 6.70 |

**Table S4.** Equilibrium lattice constants $a_{eq}$ (in A) and energy gap $E_g$ (eV) (columns 2,3) for different x values. DFT band gap, valence band top (VBT) and conduction band bottom (CBB) variations (columns 4-7) together with experimental values (italic) in columns 4-7, respectively. ΔVBT /ΔCBB in columns 5,6,7 correspond to different choices of lattice constants: optimized ($a_{eq}$) or fixed at $a_{FASI\text{-}epxr.}$ (6.36 A) and $a_{FASI\text{-}DFT}$ (6.4 A).

| ***x*** | **$a_{eq}$ / $\Delta a_{eq}/a_{eq}$ [%]** | **$E_g$** | **$\Delta E_g$ th/*exp*** | **ΔVBT /ΔCBB (*expr)*** | | |
|---|---|---|---|---|---|---|
| 0 | **6.40** | 0.885 | 0/*0* | 0 | 0 | 0 |
| 0.25 | 6.385/260.3/-0.2 | 0.94 | +55/*50* | -8 (-*70*) / +47 (-*20*) | -31 / +41 | -67/ +21 |
| 0.5 | 6.379/-0.3 | 1.04 | +159/*130* | -71(*-120*)/+88 *(+10)* | -95 / +97 | -83/ +97 |
| 0.7 | 6.36/-0.6 | 1.072 | +187 | - | -121/ +129 | -129/ +123 |
| 1 | **6.335** / -1 | 1.162 | +277/*230* | -104 (-*160*)/+173 (+*70*) | -192 / +184 | -200 / +185 |

**Table S5.** Photovoltaics performance parameters of $FASnI_{3-x}Br_x$ with varying the Br content under AM 1.5G illumination.

| **Br content (*x*)** | **$V_{oc}$ (V)** | **$J_{sc}$ (mA/cm$^2$)** | **FF (%)** | **PCE (%)** |
|---|---|---|---|---|
| 0 | 0.58 (0.58) | 19.2 (19.2) | 67 (66) | 7.4 (7.3) |
| **0.2** | **0.61 (0.61)** | **19.0 (18.9)** | **70 (70)** | **8.1 (8.0)** |
| 0.5 | 0.63 (0.63) | 17.0 (17.0) | 69 (65) | 7.4 (7.0) |
| 0.8 | 0.65 (0.65) | 13.0 (13.0) | 72 (70) | 6.1 (6.0) |
| 1 | 0.72 (0.72) | 11.6 (11.6) | 68 (68) | 5.7 (5.7) |

**Table S6.** Photovoltaics performance parameters of $FASnI_{3-x}Br_x$ with varying the Br content under 1000 lx illumination.

| **Br content (*x*)** | **$V_{oc}$ (V)** | **$J_{sc}$ (mA/cm$^2$)** | **FF (%)** | **PCE (%)** |
|---|---|---|---|---|
| 0 | 0.36 | 0.1 | 69 | 8.6 |
| 0.2 | 0.37 | 0.1 | 70 | 8.7 |
| 0.5 | 0.40 | 0.09 | 72 | 8.7 |
| 0.8 | 0.47 | 0.08 | 72 | 8.9 |
| **1** | **0.58** | **0.08** | **72** | **11.1** |

**Table S7.** Photovoltaics Parameters of $FASnI_2Br$ under different light intensities of two different WLEDs of different colour temperatures.

| CT of LEDs | Light Intensity (lx) | $V_{oc}$ (V) | $J_{sc}$ (mA/cm$^2$) | FF (%) | PCE (%) |
|---|---|---|---|---|---|
| 2900 K | 1000 | 0.58 | 0.08 | 72 | 11.1 |
| | 500 | 0.56 | 0.04 | 71 | 11.0 |
| | 200 | 0.52 | 0.02 | 72 | 10.0 |
| 3600 K | 1000 | 0.58 | 0.07 | 72 | 9.6 |
| | 500 | 0.55 | 0.04 | 72 | 9.3 |
| | 200 | 0.52 | 0.01 | 72 | 8.9 |

## References


(1) Kresse, G.; Joubert, D. From Ultrasoft Pseudopotentials to the Projector Augmented-Wave Method. *Phys. Rev. B* **1999**, *59* (3), 1758.

(2) Kresse, G.; Hafner, J. Ab Initio Molecular-Dynamics Simulation of the Liquid-Metal–Amorphous-Semiconductor Transition in Germanium. *Phys. Rev. B* **1994**, *49* (20), 14251.

(3) Kresse, G.; Hafner, J. Ab Initio Molecular Dynamics for Liquid Metals. *Phys. Rev. B* **1993**, *47* (1), 558.

(4) Kresse, G.; Furthmüller, J. Efficient Iterative Schemes for Ab Initio Total-Energy Calculations Using a Plane-Wave Basis Set. *Phys. Rev. B* **1996**, *54* (16), 11169.

(5) Kresse, G.; Furthmüller, J. Efficiency of Ab-Initio Total Energy Calculations for Metals and Semiconductors Using a Plane-Wave Basis Set. *Comput. Mater. Sci.* **1996**, *6* (1), 15–50.

(6) Kresse, G.; Hafner, J. Norm-Conserving and Ultrasoft Pseudopotentials for First-Row and Transition Elements. *Journal of Physics: Cond. Matter* **1994**, *6* (40), 8245.

(7) Perdew, J. P.; Burke, K.; Ernzerhof, M. Generalized Gradient Approximation Made Simple. *Phys. Rev. Lett.* **1996**, *77* (18), 3865.

(8) Grimme, S.; Antony, J.; Ehrlich, S.; Krieg, H. A Consistent and Accurate Ab Initio Parametrization of Density Functional Dispersion Correction (DFT-D) for the 94 Elements H-Pu. *J. Chem. Phys.* **2010**, *132* (15), 154104 .
